# Mycosynthesis of zinc sulfide/zinc oxide nanocomposite using *Fusarium oxysporum* for catalytic degradation of methylene blue dye, antimicrobial, and anticancer activities

**DOI:** 10.1038/s41598-024-81855-4

**Published:** 2024-12-31

**Authors:** Hosam Salaheldin, Aya Aboelnga, Ashraf Elsayed

**Affiliations:** 1 Biophysics Research Group, Faculty of Science, Physics Department, Mansoura University, Mansoura, 35516 Egypt; 2 Faculty of Science, Botany Department, Mansoura University, Mansoura, 35516 Egypt

**Keywords:** ZnO/ZnS bimetallic NPs, Mycosynthesis, *Fusarium oxysporum*, And antimicrobial activity, Biophysics, Nanoscience and technology

## Abstract

In the present study, extracellular cell-free filtrate (CFF) of fungal *Fusarium oxysporum* f. sp. *cucumerinum* (FOC) species, was utilized to biosynthesize zinc oxide /zinc sulfide (ZnO/ZnS) nanocomposite. This was achieved by mixing the metal salt with the fungal CFF for 96 h at a temperature of 27 ± 1.0 °C and a pH of 6.5. Several analytical techniques, such as XRD, TEM, UV-Vis, FTIR, DLS, and zeta potential studies, have confirmed the synthesis of NPs. Fungal CFF enzymes and metabolites stabilized produced NPs, according to FTIR. The nanocomposite particle diameter (15–80 nm) was estimated using HR-TEM imaging. The DLS and XRD measurements verified those findings. The zone of inhibition diameter for *MRSA* was 35 ± 0.21 mm, while *B. subtilis* measured 33 ± 0.32 mm against the nanocomposite. For *E. coli* and *S. typhi* bacterial isolates, it was 25 ± 0.19 and 32 ± 0.36 mm, respectively. The determined MIC value for *E. coli* was 5,000 µg/mL and *MRSA* was 500 µg/mL. The ZnO/ZnS nanocomposite has a dose-dependent cytotoxic effect on breast cancer cells, with an IC_50_ of 197 ± 0.895 µg/mL. The Methylene blue dye was removed by 87.51% using the nanocomposite. Thus, green biosynthesized ZnO/ZnS nanocomposites are recommended for pharmaceutical, industrial, and biological applications.

## Introduction

Nanoparticles (NPs) have been utilized in various fields, such as photocatalysis^1^, solar cells^2^, gas sensing^3^, photoluminescence materials^4^, and optoelectronics^5^. The physical, chemical, and biological techniques have all been successfully used to create a variety of nanoparticles^6,7^. The NPs can be synthesized via traditional physical and chemical^8^ processes that typically involve the utilization of hazardous substances and result in the production of waste, hence posing a risk of environmental contamination^9,10^. Therefore, green synthesis methods are progressively replacing physical and chemical methods due to the problems associated with the consumption of significant amounts of energy, the release of toxic and harmful chemicals, and the use of specialized equipment and synthesis conditions^11^. Sustainable and scalable methods for NPs production have emerged as viable alternatives, including plant, fungal, and algal-mediated synthesis^12^.

Zinc oxide (ZnO), zinc sulfide (ZnS), and ZnO/ZnS nanocomposite were synthesized via various techniques, including hydrothermal methods^13^, precipitation methods^14^, combustion methods^15^, simple thermal treatment methods^16^, and green synthesis employing biological treatment^17^. The ZnO and ZnS NPs are biosynthesized employing the usage of bioactive compounds extracted from the organisms, including plants^18,19^, bacteria^20–22^, fungi^23–25^, and yeast^26–28^. These microbial species are highly affecting the biological synthesis of metal and metal oxide NPs. Due to this, a growing interest in the use of microbes has been observed in the last decade, because of which several researches have been conducted using different microbiological models. On the other hand, however, the biogenesis of ZnO NPs by microorganisms is an issue that is unsolved to date^29^.

Fungal species, in comparison to bacteria, have a number of advantages during bioprocessing, especially when it comes to the production of gold (Au) NPs^30^. Fungi secrete large amounts of extracellular proteins used for various biological functions^31,32^. The secretome is the designation of a large group of proteins secreted into the extracellular surroundings. The homologous and heterologous proteins can be produced by highly abundant fungal secretomes. According to Kirkland et al.^31^ fungal hydrophobin from the entomopathogenic fungus *Beauveria bassiana* is one of the representative protein secretion examples used in large-scale industrial applications^31^.

The fungi have been the primary biological agents for the production of several types of NPs. Mycosynthesis is generally applied to refer to the production of nanoparticles using fungal species, one such approach that generally comes under the umbrella of myconanotechnology. *F. oxysporum* is one of the most cosmopolitan species within the Kingdom of fungi due to its inclusion of plant and human pathogens. It typically infects a wide variety of species in a host-specific manner. In general, *F. oxysporum* has been utilized in the synthesis of various metallic silver (Ag) NPs^33–35^, Au NPs^36^, ZnO NPs^37–39^, and ZnS NPs^40,41^ using extracellularly and/or intracellularly approaches.

In intracellular synthesis, the metal precursor is introduced to the mycelial culture and then internalized within the biomass. Therefore, the extraction of NPs is necessary post-synthesis, utilizing chemical treatment, centrifugation, and filtration to disintegrate the biomass and liberate the nanoparticles^42^. In extracellular synthesis, the metal precursor is introduced to the aqueous filtrate containing solely fungal macromolecules, leading to the synthesis of free NPs in the filtrate solution^43^. This is due to the presence of primary and secondary metabolites. Therefore, the extracellular approach is the most prevalent method, as it necessitates no processes for extracting the NPs from the cells^43–45^. Also, this synthesis method is an ecologically rapidly benign process compared to physical and chemical methods. Additionally, to the best of the authors’ knowledge, although there are many literature reports on the synthesis of ZnS and ZnO NPs, this is the first report on the synthesis of ZnO/ZnS nanocomposites using *F. oxysporum* fungal CFF.

In this study, we report the biosynthesis and characterization of ZnO/ZnS nanocomposite using fungal *F. oxysporum* extracellular cell-free filtrate as a reducing and stabilizing agent. The inhibitory effect of biosynthesized nanocomposite was investigated against pathogenic Gram-positive bacteria and Gram-negative bacteria. Moreover, breast cancer cell cytotoxicity activity and Methylene blue dye catalytic degradation efficacy were examined against the green synthesized ZnO/ZnS nanocomposite.

## Materials and methods

### Materials

Zinc sulfate (ZnSO_4_.7H_2_O; 99.5%) was purchased from Merck, India. Methylene blue (MB) was purchased from Central Drug House (CDH, India). Deionized water was used throughout the experiment. The fungal strain *Fusarium oxysporum f. sp*. *cucumerinum* (FOC) species was collected from a mycological laboratory at the Botany Department, Faculty of Science, Mansoura University, Egypt. The fungal isolate was cultured in potato dextrose agar (PDA) broth media (Merck, USA). The antimicrobial activity was evaluated via the use of four clinical bacterial isolates, *Bacillus subtilis* (*B. subtilis) and methicillin-resistant Staphylococcus aureus* (*MRSA)*, which are Gram-positive bacteria, whereas *Escherichia coli* (*E. coli)* and *Salmonella typhi* (*S. typhi) are* Gram-negative bacteria. The other microbe was a fungal isolate of *C. albicans*. The antibiotics were bought from Invitrogen, USA; Dulbecco’s modified Eagle’s medium (DMEM), glutamine, and sodium pyruvate from Invitrogen, U.K. MDA-MB-231 cancer cell lines were obtained from the molecular biology unit, Faculty of Science, Mansoura University. Fetal calf serum (FCS), 3-(4,5-dimethylthiazol-2-yl)-2,5-diphenyltetrazolium bromide (MTT), sodium hypochlorite solution (NaOCL, accessible chlorine 10–15%), and dimethyl sulfoxide (DMSO) were given from Sigma-Aldrich, USA. Every chemical used in the tests was graded as an analytical reagent.

### Methods

#### Biosynthesis of ZnO/ZnS nanocomposite by *F. Oxysporum*

For the biosynthesis of the NPs, *F. oxysporum* was cultured in 250 mL PDA broth at 25 ± 1.0 °C for 5 days under shaking conditions (180 revolutions per minute; rpm). For cell autolysis, the biomass was centrifuged at 5,000× g for 15 min (Thermo Scientific, USA), rinsed three times with sterile distilled water, and resuspended in sterile water for four days. Further, the autolysate was centrifuged at 4000× g for 5 min to obtain the cell filtration, which was subsequently filtered through sterile Whatman filter paper No. 1. The resulting liquid portion, classified as a supernatant (CFS) and a cell-free filtrate (CFF), was employed in the biosynthesis of ZnO/ZnS nanocomposite.

**Fig. 1:**
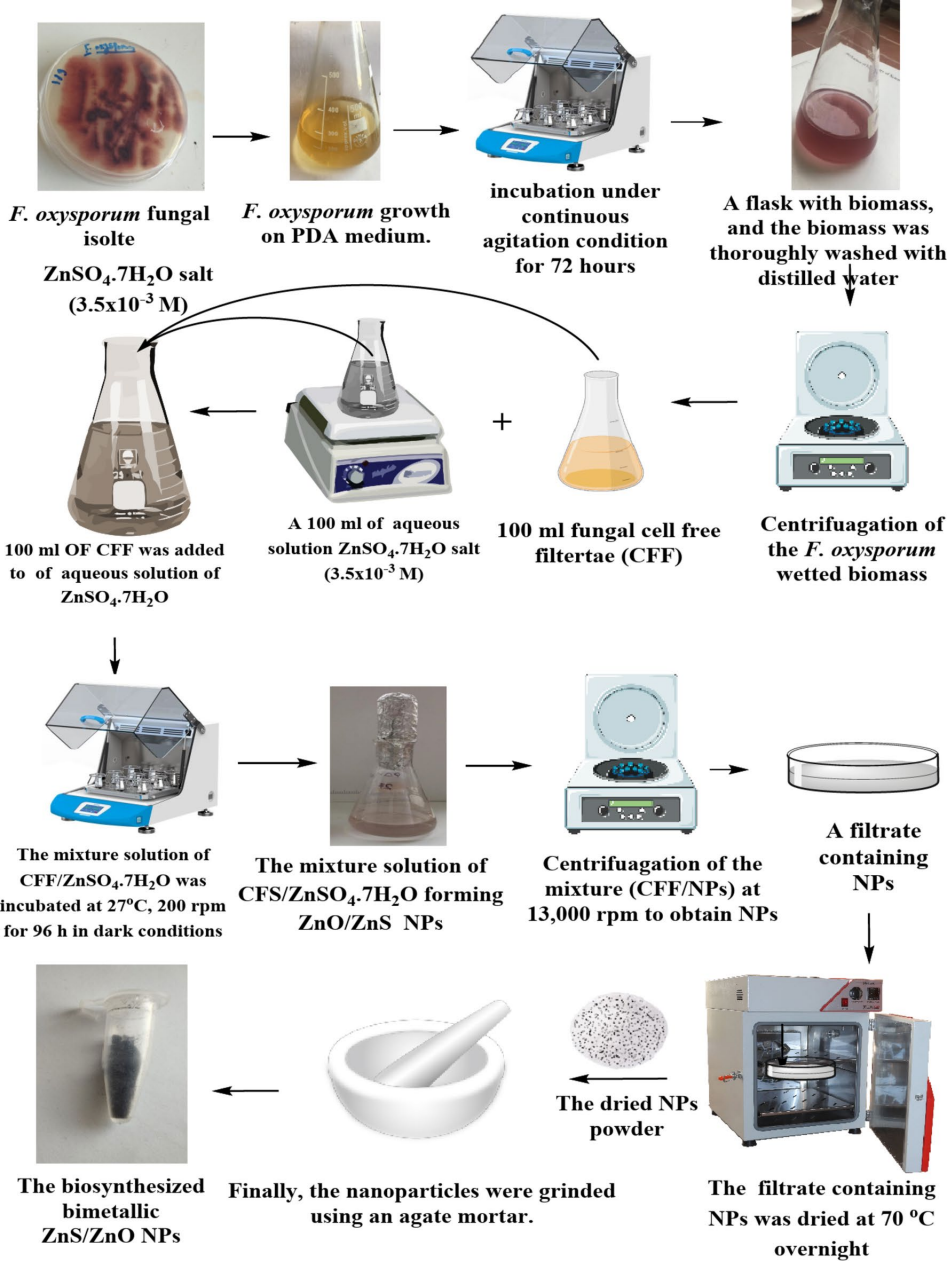
Schematic representation of the mycosynthesis of ZnO/ZnS nanocomposite utilizing *F. oxysporum.*

The mycosynthesis of ZnO/ZnS nanocomposite involved treating the fungal extract derived from *F. oxysporum* with 100 mL aqueous solution of ZnSO_4_.7H_2_O (3.5 mM final concentration). In the ZnO/ZnS nanocomposite synthesis process, the temperature of the mixture of CFF and ZnSO_4_.7H_2_O (1:1, v/v) was adjusted to ambient room temperature (27 ± 1.0 °C). Thereafter, the mixture was incubated at 27 ± 1.0 °C and a rotational speed of 200 rpm for 96 h in the dark (Fig. 1). Finally, the biosynthesized ZnO/ZnS nanocomposite was centrifuged at 13,000 × g for 10 min (Hettich Mikro 20 Centrifuge, Germany). The sample was thoroughly rinsed three times with deionized water to eliminate impurities. After washing, these pellets underwent a 12 h cycle of oven drying at a temperature of 70 °C. The obtained pellets were finely ground into powder by using agate mortar following the drying procedure to ensure the fine dimensions of the obtained nanocomposite (Fig. 1).

#### Characterization of the biosynthesized ZnO/ZnS nanocomposite

The physicochemical properties of ZnO/ZnS nanocomposite were quantified by the following methods. The UV-Vis spectrophotometer (Jenway 7205, United Kingdom) was employed to ascertain the ultraviolet-visible (UV-Vis) absorption spectrum at room temperature over the 300–800 nm wavelength range. Deionized water was initially used as a blank reference^46^.

Fourier Transform Infrared (FTIR) spectroscopy, which is based on the vibration and rotation of atoms, is a ubiquitous and extensively used spectral equipment for identifying internal molecular structures in many fields^47^.

A JASCO FT-IR 4100 spectrometer was used to analyze FT-IR patterns to investigate possible biomolecules present in the biosynthesized ZnO/ZnS nanocomposite. The samples weighing 0.2 g were mixed with potassium bromide (KBr) and subsequently compressed into discs under high pressure. In transmission mode, infrared spectra were acquired by conducting scans with a resolution of 4.0 cm^− 1^ across the 400–4000 cm^− 1^ range.

The X-ray diffraction (XRD) pattern of the ZnO/ZnS nanocomposite was analyzed, using X’Pert Pro X-ray diffractometer produced (Philips Eindhoven, Netherlands). The apparatus was fitted with a proportional counter and employed nickel-filtered copper K-alpha radiation with a wavelength (λ) of 1.5405 Å. The XRD measurements were carried out at 40 kilovolts (kV) and a current of 30 milliamperes (mA). The crystalline structure of the ZnO/ZnS NPs was analyzed throughout a 2θ range extending from 10^°^ to 80^°^.

The size of mycosynthesized nanocomposite in liquid solution was determined using Zetasizer nanodevices, Zetasizer Nano ZS, Malvern, UK, through the DLS technique. Samples were prepared by suspending nanoparticles in de-ionized water at 25 ppm. The suspension was then vortexed for homogeneity, and 1.5 ml of the solution was put into a square cuvette for subsequent measurements. Zeta potential was determined using a Zetasizer nanoarray (HT Laser, ZEN 3600; Malvern Instruments, UK).

Transmission electron microscopy (TEM) (model JEOL TEM-2100, USA) was used in the measurement of the size and shape of nanocolloidal samples. A carbon-coated grid Type G 200, with a diameter of 3.05 prepared by^48^. EDX studies were conducted on Shimadzu DX-700HS equipment to identify and quantify coupled with a scanning electron microscopy (SEM) instrument (JEOL JSM 6510 lv, USA) and an energy-dispersive spectrometer detector.

### Antimicrobial activity test

#### Antimicrobial activity

The biosynthesized *F. oxysporum* CFF-ZnO/ZnS -nanocomposite were tested for their antimicrobial effect against several Gram-positive and Gram-negative bacteria, such as *B. subtilis*,* MRSA*, *E. coli*, and *S. typhi*, and fungal isolate *C. albicans*using the well diffusion method^49,50^. The Petri dishes were sterilized by autoclaving at 121 °C for 20 min. A similar procedure was followed for the preparation and autoclaving of Luria bouillon. Afterward, L.B. agar was incorporated, and 20 mL of the agar medium was evenly spread to a depth of 4 mm. Pure pathogenic bacterial strains at 10^7^ colony-forming units (CFU/mL) were streaked on the prepared agar plates individually using aseptic cotton swabs and left for 18 h^51^. Using a cork borer, wells were then created in the agar, into which suspensions of ZnO/ZnS nanocomposites were added at concentrations of 50, 100, and 150 µg/mL. To evaluate the zone of inhibition (ZIs), the Petri dishes were incubated for 24 h at 37 ± 1.0 °C. The test was conducted in triplicate, and the ZI was measured using a ruler (AIM^®^) and a pair of dividers then results were reported in millimeters (mm).

### Minimum inhibition concentration (MIC) assay

To evaluate the MIC of the ZnO/ZnS nanocomposite against the tested microbes, variable concentrations of nanocomposite (50, 500, 5000, and 50,000 µg/mL) were carefully transferred into test containers. Each injected with 100 µL of a sterilized standard inoculum specifically prepared for the microbial species using L.B. broth medium^49,52^. The bottles, now containing the bacterial species, were incubated at 37 ± 1.0 °C for 24 h to facilitate optimal growth. For the control containers, both growth medium and inoculum were added for each microorganism examined. The bottles that displayed no turbidity compared to the control sample were utilized to determine the MIC. The MIC values indicate the concentration of the sample (in µg/mL) required to effectively inhibit microbial growth, highlighting the significant antibacterial properties of the nanocomposite.

### Scanning electron microscopy (SEM) imaging

SEM is an appropriate instrument for examining the microstructure and morphology of bacterial cells. To evaluate the efficacy of ZnO/ZnS nanocomposite against bacterial cells that have been untreated and treated. SEM (JEOL JSM 6510 lv, USA) micrographs were conducted to examine the effect of the ZnO/ZnS nanocomposite against *E. coli* and *MRSA* bacterial isolates.

### MTT cell cytotoxicity assay

MDA-MB-231 cells were subjected to treatment with varying concentrations of ZnO/ZnS nanocomposite at 500, 250, 125, 62.5, 31.25, and 15.62 µg/mL for 24 h to evaluate cytotoxic effects. Following the treatment, 10 µL of MTT solution (5 mg/mL) was introduced to each well and incubated at 37 ± 1.0 °C for 30 min. After incubation, absorbance was measured at 540 nm with a multi-well plate reader following the addition of 100 µL of DMSO to dissolve the purple precipitate produced by formazan. The viability of the cells was evaluated by comparing the proportion of viable treated cells to that of untreated cells.

1 $${\text{Cell proliferation inhibition}}\:\% = \:\frac{{{\text{Mean absorbance of the ZnO/ZnS nanocomposite - Mean absorbance of the sample}}}}{{{\text{Mean absorbance of the control - Mean absorbance of the sample}}}} \times100.$$

The 50% inhibitory concentration was determined via a dose-responsive curve for the ZnO/ZnS nanocomposite (IC_50_). Based on the IC_50_ results, the ideal concentrations for subsequent studies were selected.

### Methylene blue (MB) dye degradation

To study the MB dye degradation, MB dye powder was dissolved in deionized water to make a 1000 µg/mL stock solution^39^, which was the necessary amount to do this. A dilution was performed to achieve the desired concentration (50 ppm) by mixing 5 ml of a stock solution with 95 ml of distilled water^39^. The final solution constituted 0.01 mg of ZnO/ZnS nanocomposite. The quantity of MB in the supernatant was determined at a wavelength of 664 nm utilizing a double-beam UV-Vis spectrophotometer, before and after adsorption and or/ degradation. To monitor the MB dye degradation, a variety of time intervals were employed, including 0, 5, 10, 15, 30, 60, 120, and 180 min.

### Statistical analysis

The data collected were evaluated using statistical analysis with a paired t-test through the IBM SPSS Statistics 20.0 software package. All experimental tests were conducted in triplicate, and the results were presented as mean ± SD. The *p*-value was less than 0.05 (*p* < 0.05) giving that the difference between the groups was statistically significant.

## Results and discussion

### Characterization of the ZnO/ZnS nanocomposite

#### UV‒vis spectroscopy

**Fig. 2:**
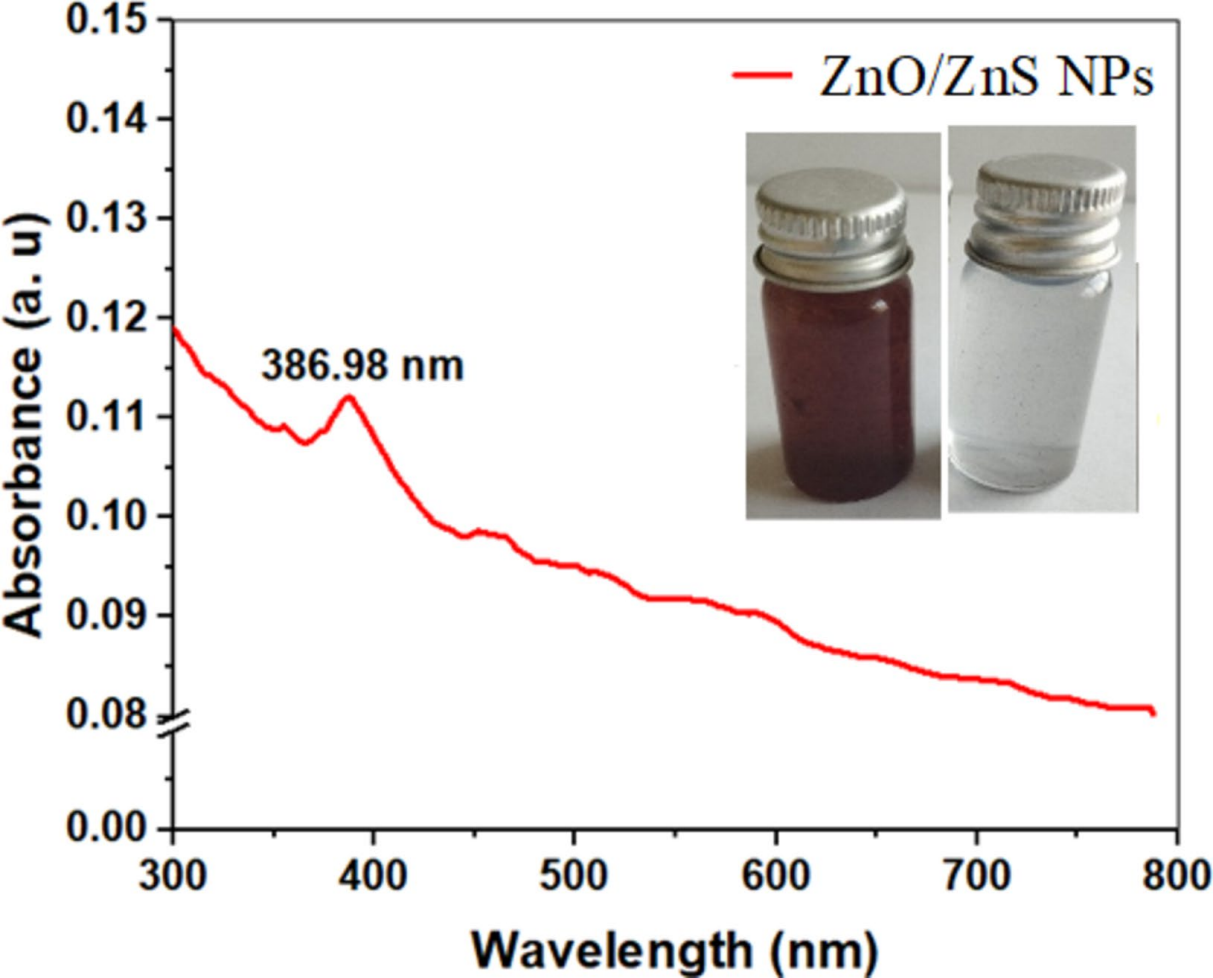
UV‒Vis absorption spectrum of the ZnO/ZnS nanocomposite biosynthesized from *F. oxysporum* dispersed in a deionized water solution; inset: digital photograph of the *F. oxysporum* CFF (left bottle) and synthesized ZnO/ZnS nanocomposite (right bottle).

The UV‒Vis absorption spectrum of the biosynthesized ZnO/ZnS nanocomposite was recorded (Fig. 2). It is widely recognized that ZnO NPs exhibit a prominent absorption peak at a wavelength of 350 nm, whereas the band edge of ZnS NPs is typically observed at approximately 345 nm^53,54^. Therefore, the absorption of a small band of the ZnO/ZnS nanocomposite was observed at approximately 385 nm^54^. However, in general, ZnO/ZnS nanocomposites display low and smooth visible light absorption due to their distinctive large energy band gap^55^ (Fig. 1). Furthermore, the apparent increase in absorption in the visible range can be ascribed to the light scattering phenomena exhibited by the patterned ZnO/ZnS nanoparticles^55^. In addition, the Gaussian absorption spectrum behavior can be attributed to the interference of light caused by the structured ZnO/ZnS NPs^56^. The impact of ZnS tied to the surface of ZnO on the absorption properties of ZnO in the visible light spectrum is significant^57,58^. Additionally, the combination of ZnS with ZnO develops a hybrid orbital within the valence band, causing a significant enhancement in the absorption of visible light. The ZnS compound contains sulfur as an anion (S^2˗^). Moreover, the valence band of the ZnO/ZnS complex is potentially dominated by S^3^p orbitals because of their comparatively higher potential energy in relation to O^2^p orbitals. The findings indicate that the ZnO/ZnS photocatalyst demonstrates higher photocatalytic efficiency than pure ZnO when exposed to visible light^54^.

### XRD analysis

**Fig. 3:**
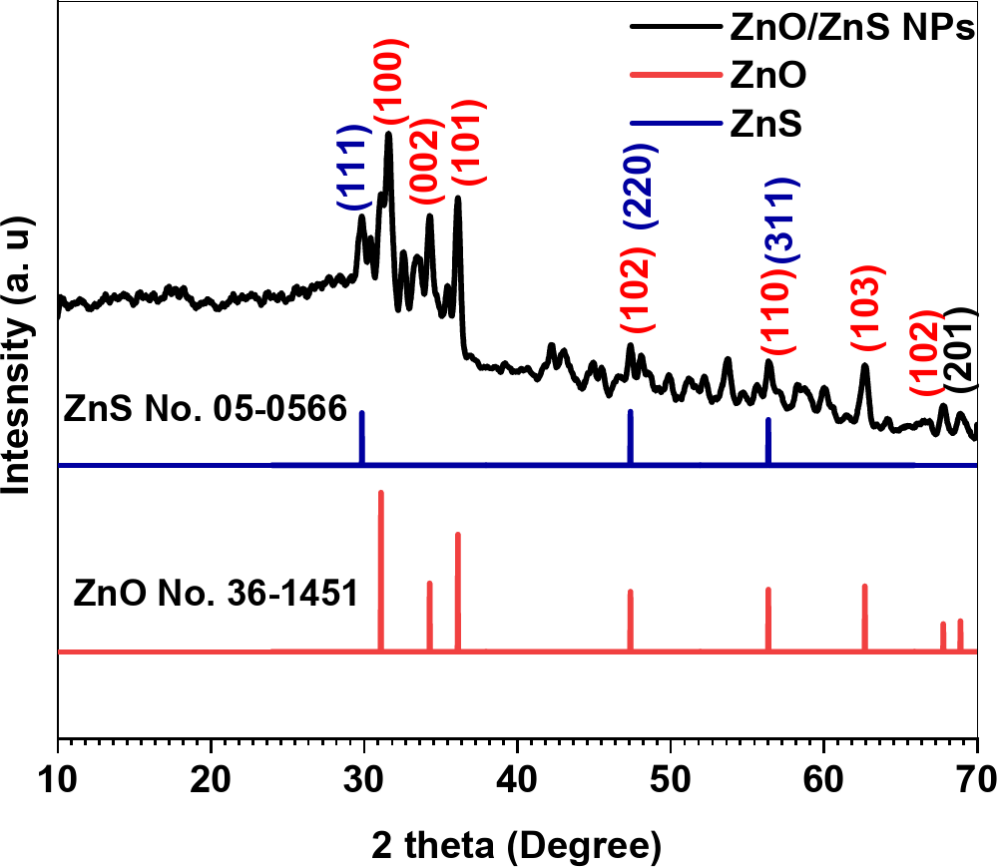
XRD pattern of the biosynthesized ZnO/ZnS nanocomposite.

The XRD pattern of biosynthesized ZnO/ZnS NPs was obtained. Compared with the standard card of ZnO NPs, JCPDS: 36-1451 standards for powder diffraction. The peaks in the ZnO pattern are related to (100), (002), (101), (102), (110), (103), (112), and (201). Moreover, there are additional peaks attributed to the ZnS NPs, according to JCPDS: 05-0566, where the peaks at 29°, 47.51°, and 56.62° belong to ZnS^59^. Furthermore, the peaks in the JCPDS ZnO and ZnS patterns are sharper than those in the ZnO/ZnS NP pattern (Fig. 3). Moreover, in the ZnO/ZnS pattern, the strength of the ZnO-related peaks decreased, and the peaks broadened as ZnS formed^60,61^. The observed decrease in the peak intensity suggests that ZnS NPs might surround and/or be adjacent to the ZnO NPs. The diffraction pattern for the ZnO/ZnS nanostructure exhibited the peak that was identified, as shown in Fig. 3.

**Fig. 4:**
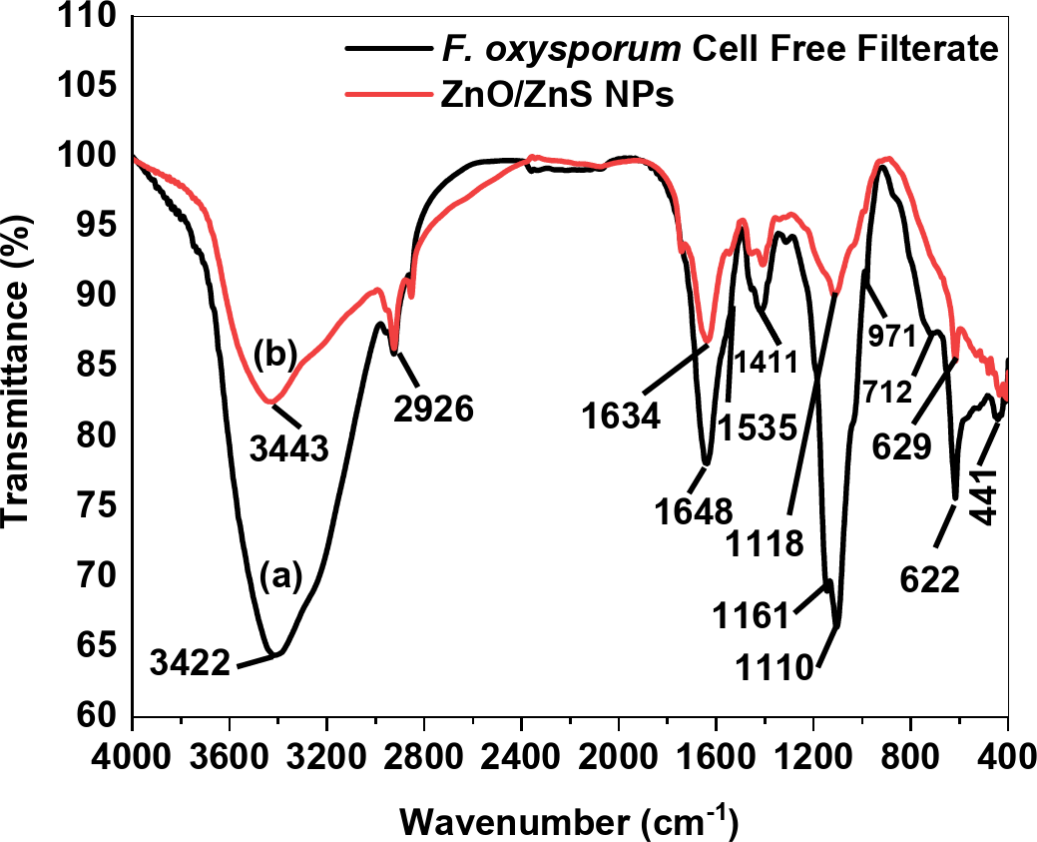
Transmittance spectra of FT-IR for *F. oxysporum* CFF (**a**) and biosynthesized ZnO/ZnS nanocomposite (**b**).

The FT-IR studies were conducted to determine the potential interactions between the ZnO/ZnS nanocomposite and *F. oxysporum* filtrate biomolecules. These molecules play pivotal roles in the synthesis, stability, and efficient dispersion of the ZnO/ZnS nanocomposite within the reaction mixture. The FT-IR spectrum exhibited prominent absorption peaks at wavenumbers of 3422.24, 2926.26, 1648.80, 1411.87, 1110.8, and 622.43 cm^− 1^, as observed in the presence of *F. oxysporum*^62^, as shown in Fig. 4. The presence of a capping agent derived from *F. oxysporum* extract in the ZnO/ZnS nanocomposite is evidenced by the observed wavenumbers at 3443.4, 2927.86, 2870.19, 1634.1, 1529.2, 1327.17, 1118, 629.91, and 471.5 cm^− 1^. Further, the spectral analysis of biogenic ZnO/ZnS nanoparticles revealed distinct peaks at wavenumbers of 3385.4, 2927.86, and 2870.19 cm^− 1^, corresponding to the stretching vibrations of O‒H bonds, C‒H bonds in aromatic compounds, and intramolecularly linked O‒H groups, respectively^37,63^. Other peaks were observed at 1634.1 cm^− 1^ and 1529.2 cm^− 1^, which correspond to the amide I band. Furthermore, two other peaks were also observed at 1327.17 cm^− 1^ and 1118 cm^− 1^, assignable to C–H bending of both aldehydes and alkanes and C–N stretching of aromatic and aliphatic amines, respectively^64,65^. In the present study, peaks, at 629.1 and 471.5 cm^− 1^ can be attributed to the formation of the Zn–O bond, as reported by^66,67^. The other absorption peak at 1194.1 cm^− 1^ was related to the presence of sulfide groups^41^. Therefore, biological molecules like proteins, amides, long-chain fatty acids, and polysaccharides are responsible for capping and maintaining nanoparticles of ZnO/ZnS, apart from facilitating the bioreduction of ZnO and ZnS ions^37,38,41^.

### EDX spectroscopy analysis

**Fig. 5:**
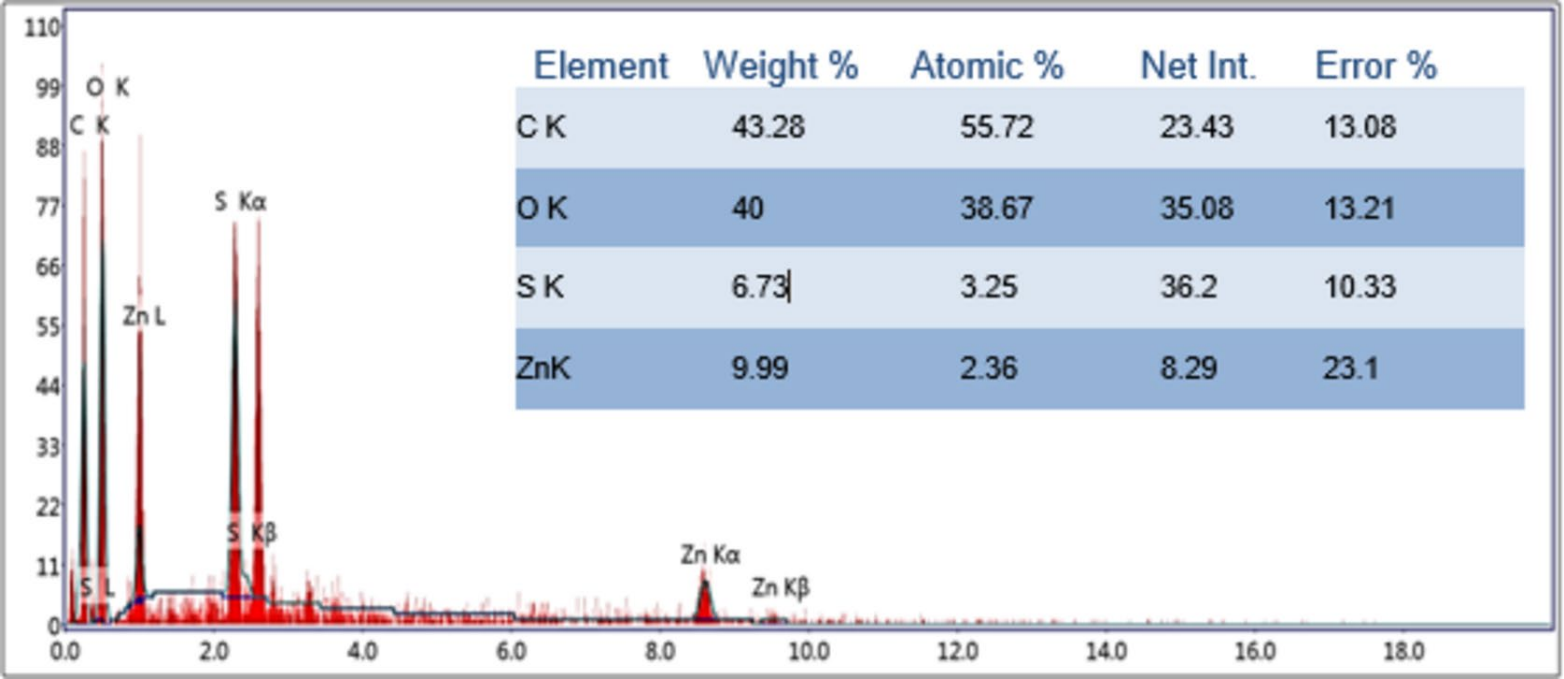
EDX spectrum of the biosynthesized ZnO/ZnS NPs.

The EDX measurements were performed to investigate the elemental composition of biosynthesized nanocomposite. The spectrum, displayed in Fig. 5, indicated the presence of the expected elements such as zinc (Zn), sulfur (S), and oxygen (O). Colpaert et al.^68^, Bakhtiari et al.^69^; and Tai et al.^70^ also reported similar elements to be present in the biosynthesized sample. In addition, a carbon peak was also detected, which might belong to the carbon residuals present in the CFF of *F. oxysporum*^45^.

### TEM analysis

**Fig. 6:**
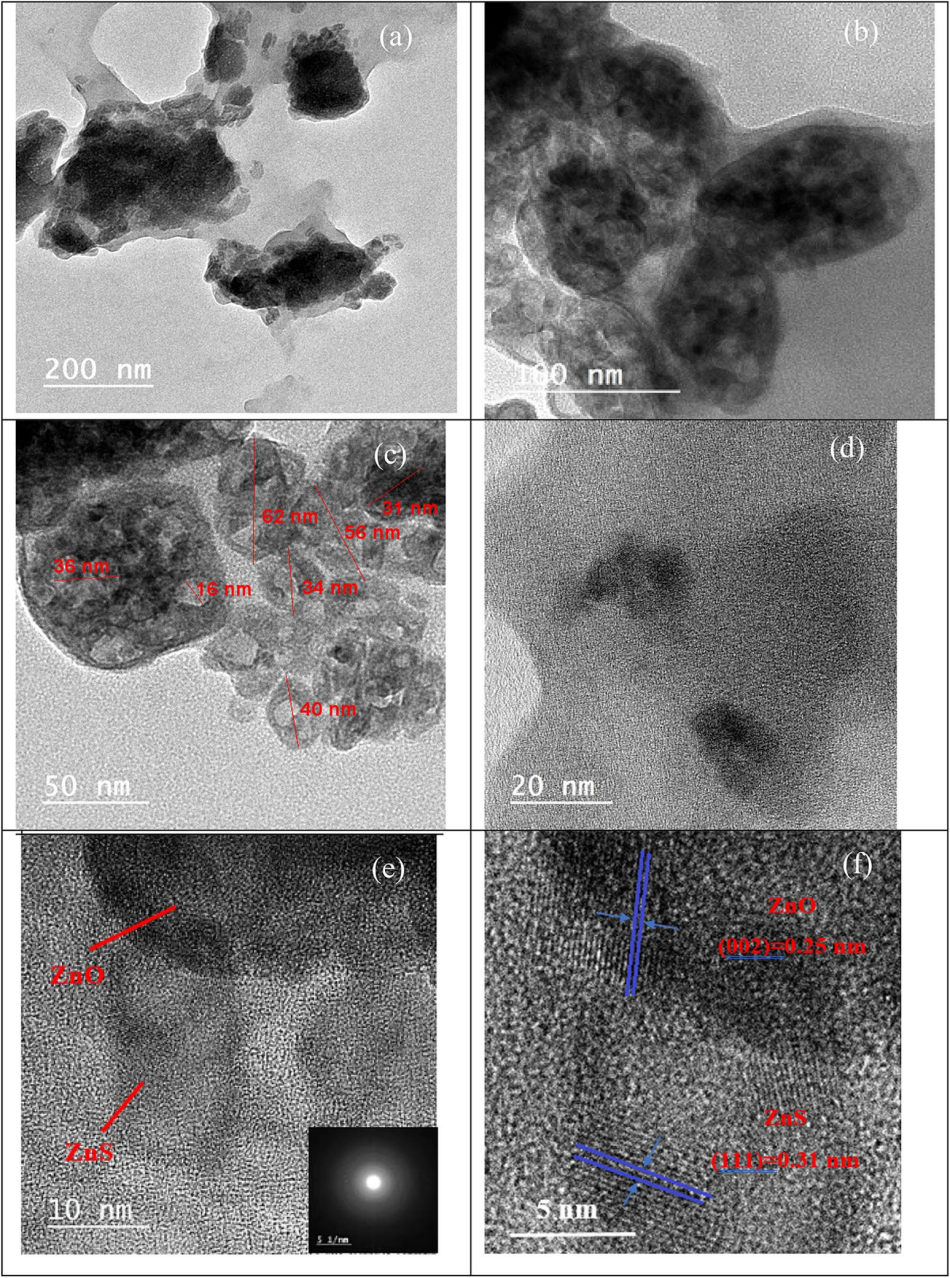
TEM micrographs of ZnO/ZnS nanocomposite synthesized from *F. oxysporum* fungal extracts (**a**–**e**) at different magnifications with an inset: SAED pattern, (**f**) HR-TEM images of ZnO/ZnS nanocomposite where the d-spacings of the ZnO and ZnS nanostructures were calculated at different locations.

Figure 6(a-d) displays HR-TEM images at various magnifications in the edge region of the ZnO/ZnS nanocomposite. The TEM image depicts the ZnO/ZnS composite, whereby a ZnS NP is adjacent to the ZnO NP sphere^71^. It can be noticed that the average ZnO/ZnS nanocrystal size was between 15 and 80 nm (Fig. 6b). Distinct and regular lattice fringes are observed in the profile of the selected area electron diffraction (SAED) pattern of the ZnO/ZnS NP pattern, as shown in Fig. 6e, which is indicative of their highly crystalline nature^55^. Furthermore, the lattice space between the (002) and (111) lattice planes of wurtzite ZnO and cubic ZnS was found 0.25 and 0.31 nm (Fig. 6f) for ZnO and ZnS, respectively^71^. These findings confirm the effective deposition of ZnS NPs beside the surface of the ZnO NPs^72^.

### Zeta potential and Zetasizer

**Fig. 7:**
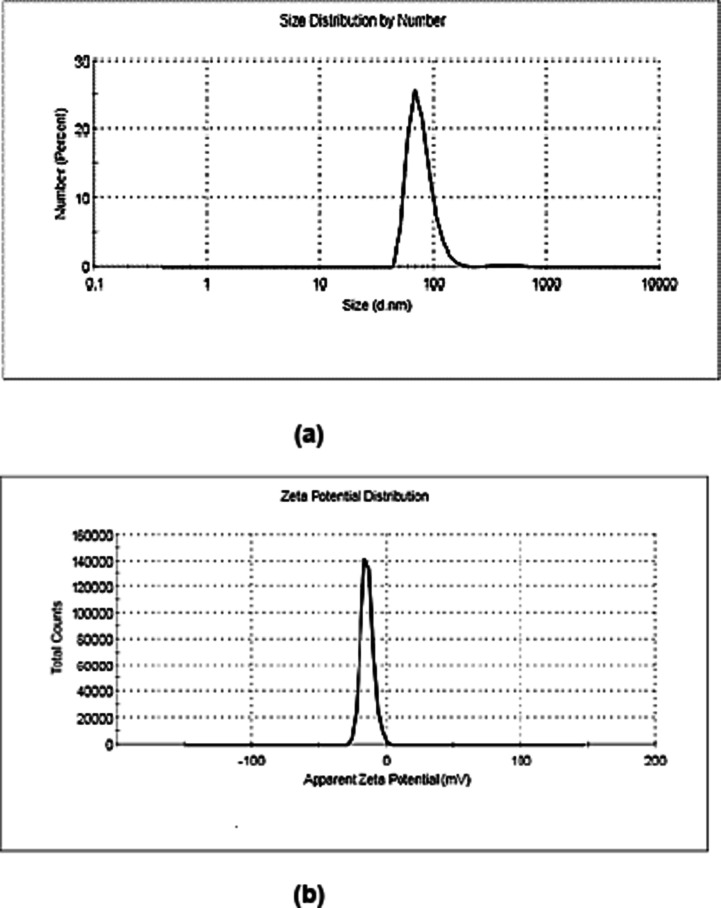
Particle size distribution spectrum of the biosynthesized ZnO/ZnS nanocomposite via DLS (**a**) and (**b**) zeta potential spectrum of the ZnO/ZnS nanocomposite.

The DLS technique was employed to assess the size distribution of the synthesized nanoparticles. The obtained findings indicate that the mean particle size diameter was found to be 77 ± 0.94 nm with a range of 30 to 110 nm (Fig. 7a). The biosynthesized ZnO/ZnS bimetallic NPs had a polydispersity index (PDI) value of 0.535, indicating that they were nearly monodisperse^62^. Furthermore, the zeta potential value, as shown in Fig. 7b, of the mycosynthesized nanocomposite was − 14.5 mV, indicating that the nanoparticles were nearly stable^62,73^.

### Scheme for the biosynthesis of ZnO/ZnS nanocomposite

**Fig. 8:**
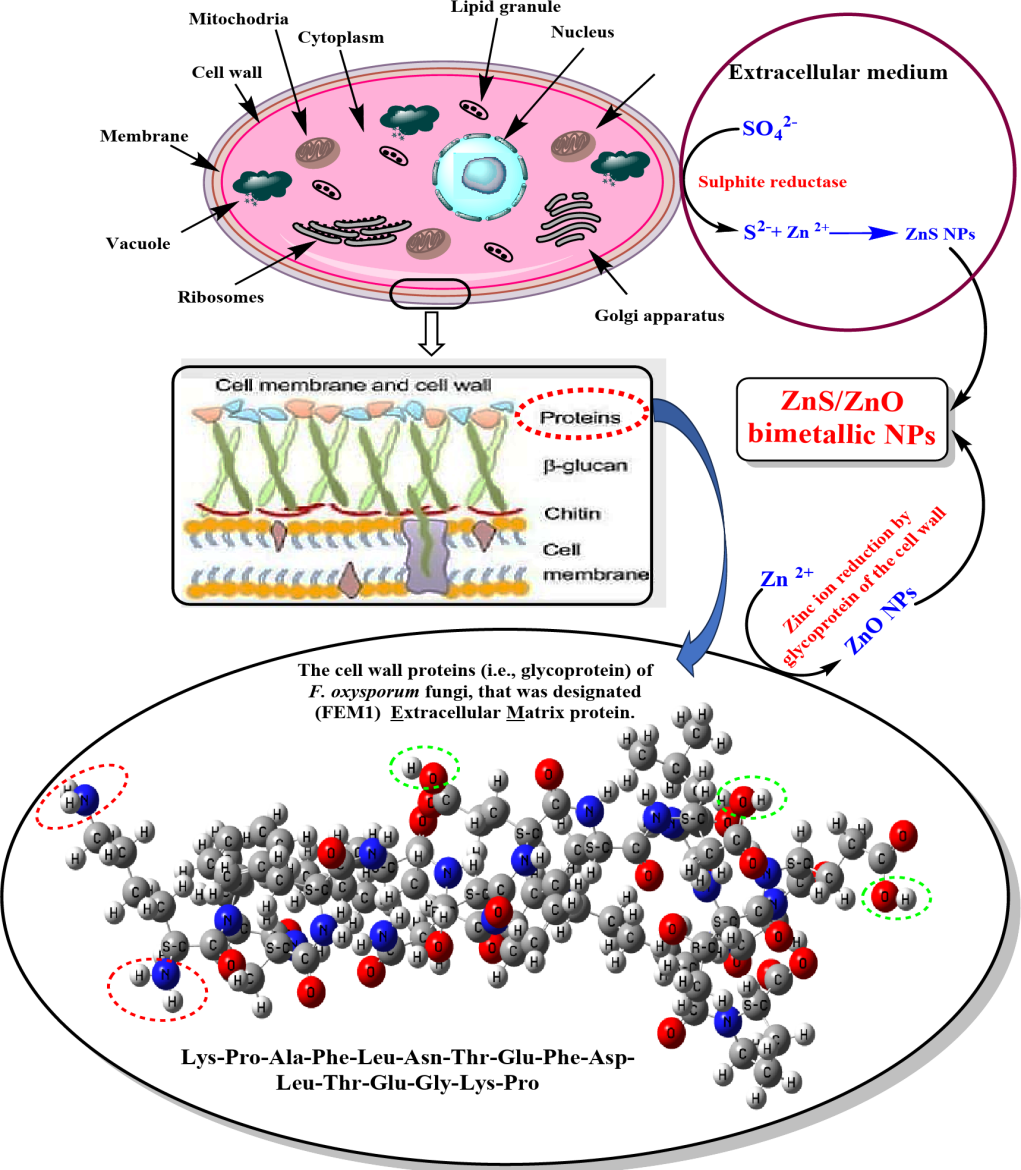
The proposed mechanism for the biosynthesis of ZnO/ZnS nanocompositeusing *F. oxysporum* FFE metabolites.

The processes through which fungi detoxify to produce nanoparticles from sources play a role in the proposed method for generating NPs using FFEs^74^. Since fungi can impact detoxification processes such as the accumulation of oxygen species (ROS), they are instrumental in these procedures^68,74^. When a metal or its compound interacts with an organism’s system, microscopic dimensions are typically addressed in nanoscale terms^75^. The endoplasmic reticulum plays a role in detoxifying metal ions that enter the cytoplasm through oxidation or oxygenation processes^76,77^. According to Priyanka^75^ and Singh et al.^78^, sulfite reductase is responsible for converting ions (SO₄²⁻) from the environment into sulfide ions (S²⁻) as the initial step in producing ZnS NPs. Figure 8 depicts ZnS nanoparticles form when zinc metal ions in a solution react with sulfide ions chemically. This process is observed in cells and tissues in general because fungal cells exhibit increased phytochelatin production when exposed to heavy metal ions.Glutathione (γGlu-Cys-Gly) phytochelatins and metallothioneins are the substances involved in the detoxification process, within fungal cells^79^. Sixteen amino acids, like Phytochelate and glutathione, are known to help the body detoxify metals through metabolic processes. These substances work within the cellular cytoplasm alongside factors, like Heavy Metal Tolerance Factor 1 (HTF I) and enzymes found in cell membranes known as membrane bound monooxygenases^78^.

In addition, biosynthesis of ZnO NPs might prove promising with fungi owing to their extraordinary resistance to high metal concentrations, extensive binding capability, and bioaccumulation capacity. Moreover, it can extracellularly synthesize quite a significant amount of redox proteins and enzymes capable of forming NPs. The reduction of metal ions extra-cellularly is done by fungal membrane glycoproteins that can also synthesize nanoparticles. Jofillmaier et al.^79^ reported that more than 15 amino acids from fungi membranal glycoproteins may be responsible for the stability of biosynthesized NPS. Furthermore, the FTIR data corroborated the correlation absorption band at 1634 cm^-1^ to the amide II band allowing C = O stretching. This was in agreement with Mohd Yusofet al.^80^. On the other hand, such processes have consequences that allow a more significant quantity of metal ions to be converted into nanoparticles hence rendering them suitable for large-scale fabrication^81^. In addition to that, the more significant amount of protein released through the fungus into the medium served as a capping agent, which further attached to and enclosed the surface of nanoparticle, hence increasing their stability^74,75^.

### Antimicrobial activity

**Fig. 9:**
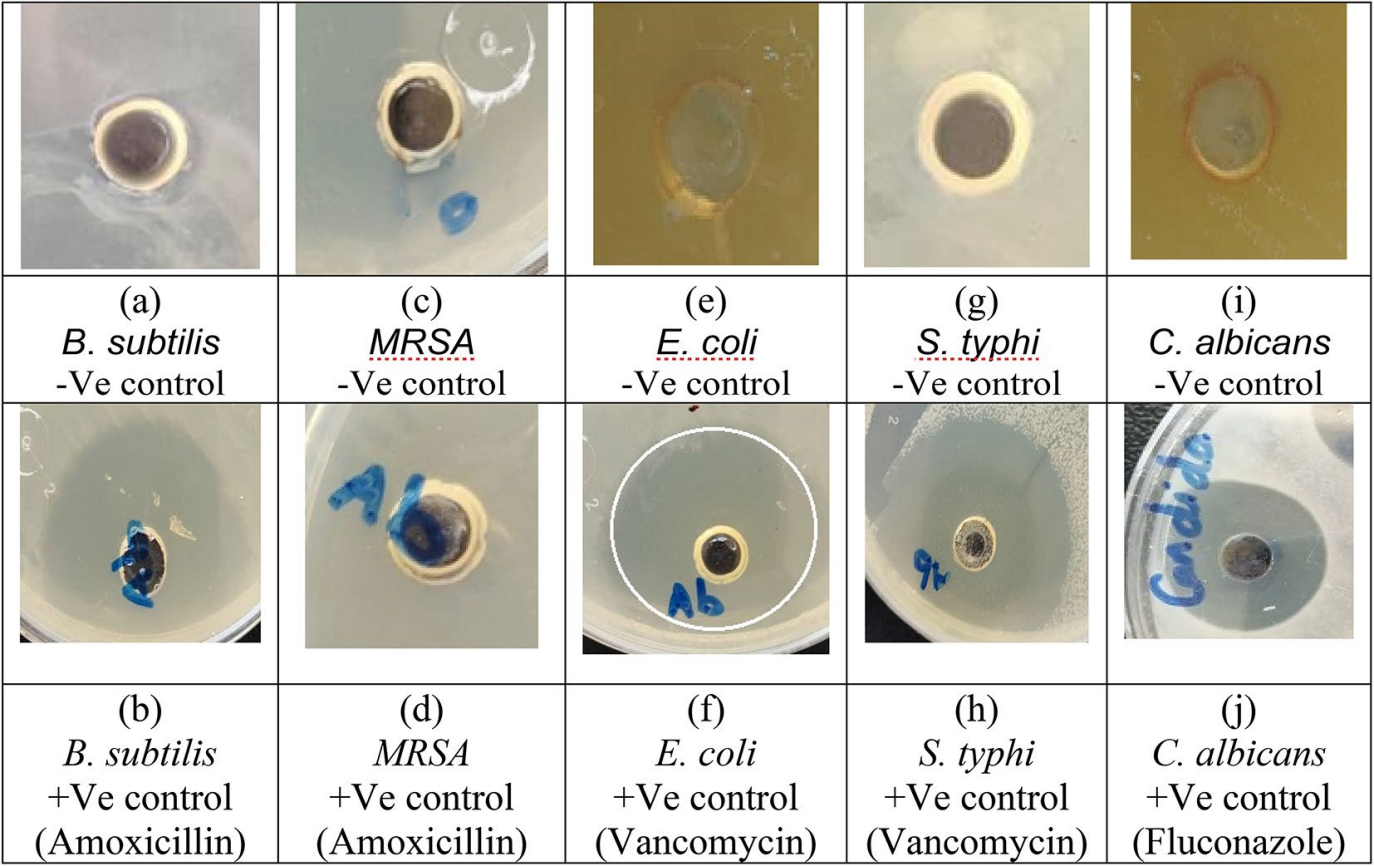
Antimicrobial activity of the + Ve control (antibiotics, i.e., Amoxicillin and Vancomycin for + Gram bacteria and -Gram bacteria, respectively) and FFE (-Ve control) samples against the pathogenic bacterial and fungal isolates.

**Fig. 10:**
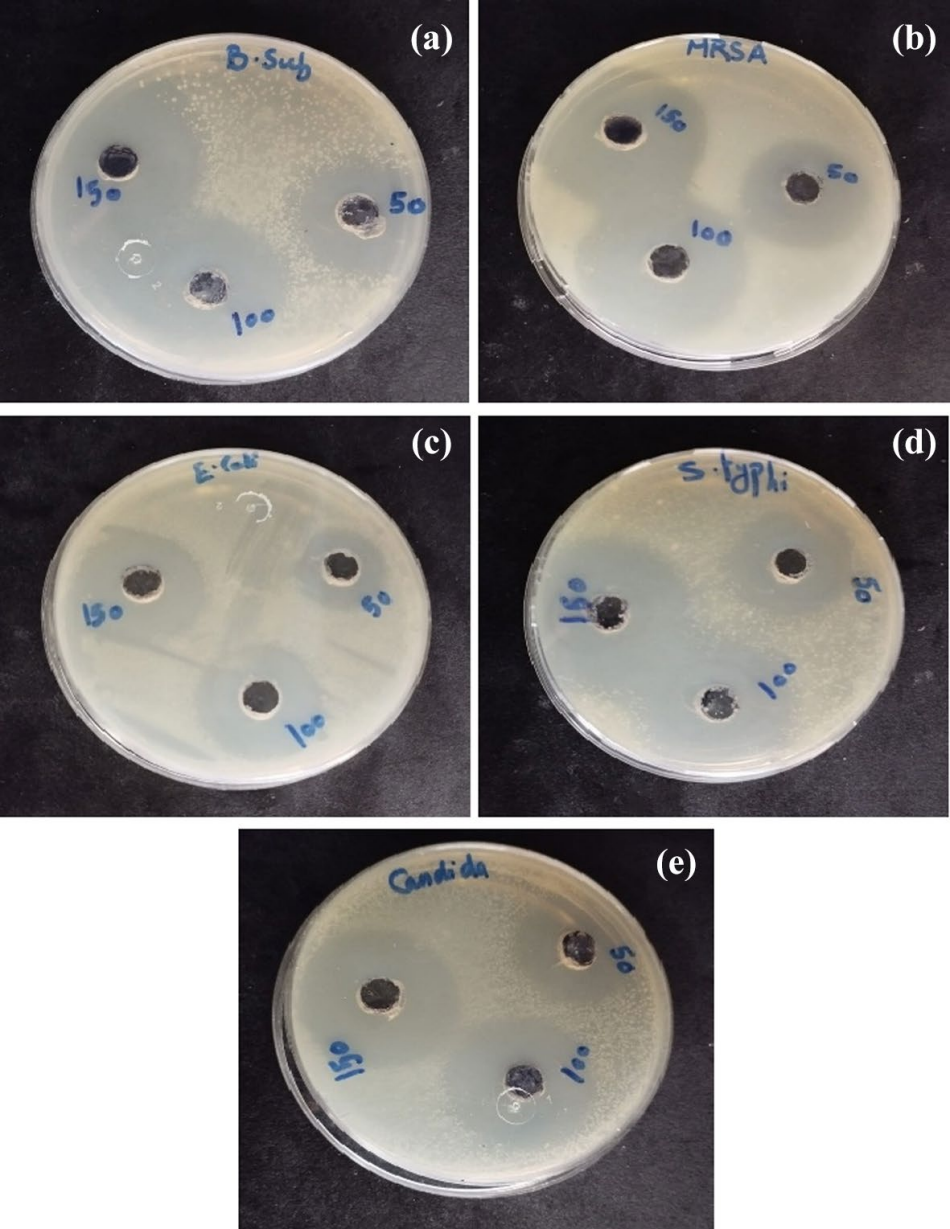
Antimicrobial activity of ZnO/ZnS nanocompositeagainst the pathogenic bacterial isolates (**a**) *B. subtilis*, (**b**) *MRSA*, (**c**) *E. coli*, (**d**) *S. typhi*, and (**d**) *C. albicans* fungal isolate.

The well diffusion method was used to evaluate the antibacterial and antifungal activities of ZnO/ZnS nanocomposite against pathogenic isolates of *B. subtilis*, *MRSA*, *E. coli*, *S. typhi*, and *C. albicans*, at different concentrations of 50, 100, and 150 µg/mL. Further, each plate was inoculated with 10^7^ CFU of either bacterial or fungal culture (for C. albicans fungal isolate). The Amoxicillin and Vancomycin antibiotics (+ Ve control) were used for + Gram and -Gram bacterial isolates. Whereas the FFEs were conducted as -Ve control for the tested bacterial and fungal isolates (Fig. 9). Additionally, the diameters of the ZIs as shown in Fig. 9e, were expressed as mean ± SD (in mm). The results of the antibacterial activity of ZnO/ZnS nanocomposite in different concentrations against the examined pathogenic *B. subtilis*, *MRSA* (Gram + Ve) isolates are illustrated in Fig. 10.

**Fig. 11:**
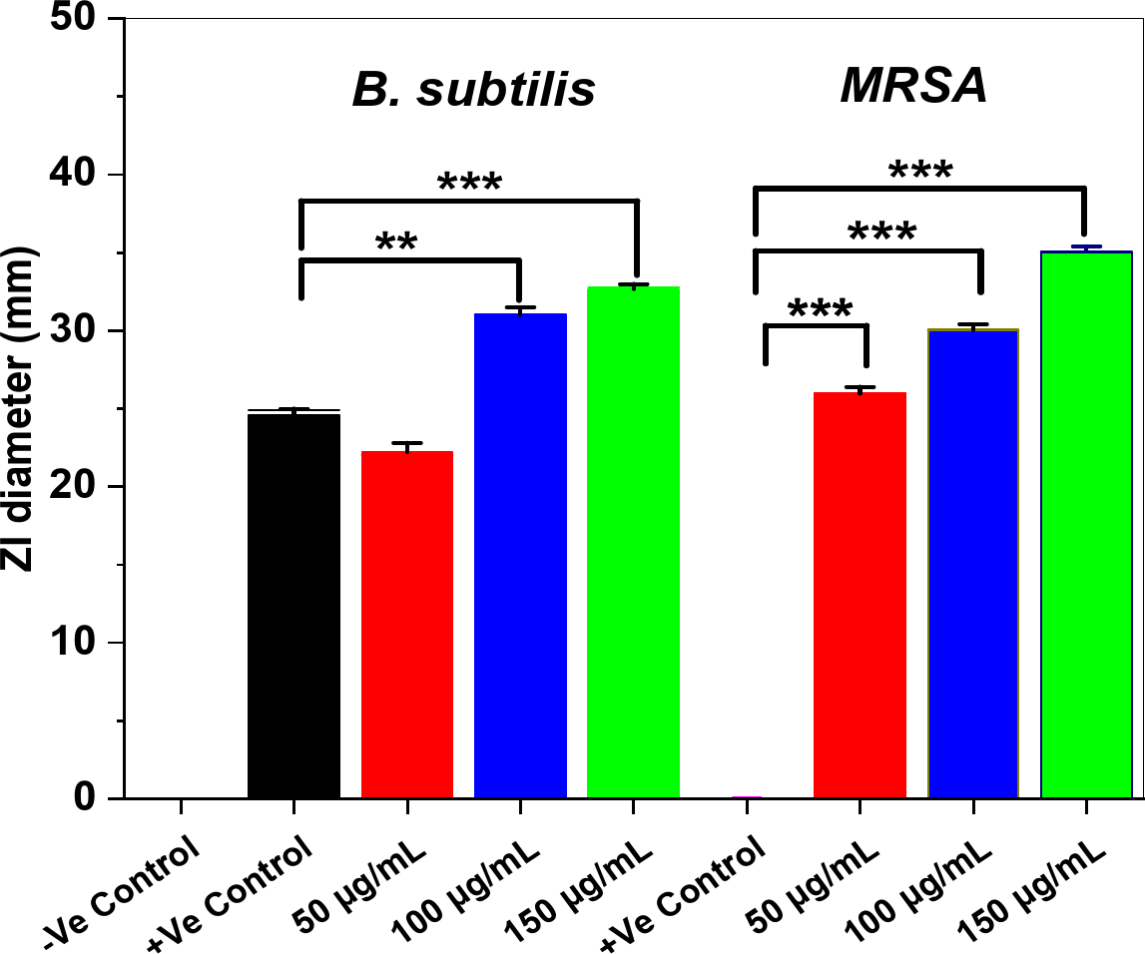
The ZI diameters with mean ± SD (in mm) of the investigated pathogenic *B. subtilis* and *MRSA* (Gram + Ve) bacterial isolates against various concentrations (50, 100, and 150 µg/mL) of ZnO/ZnS nanocomposite. *p < 0.05, **p < 0.01, ***p < 0.001 (*n* = 3).

**Fig. 12:**
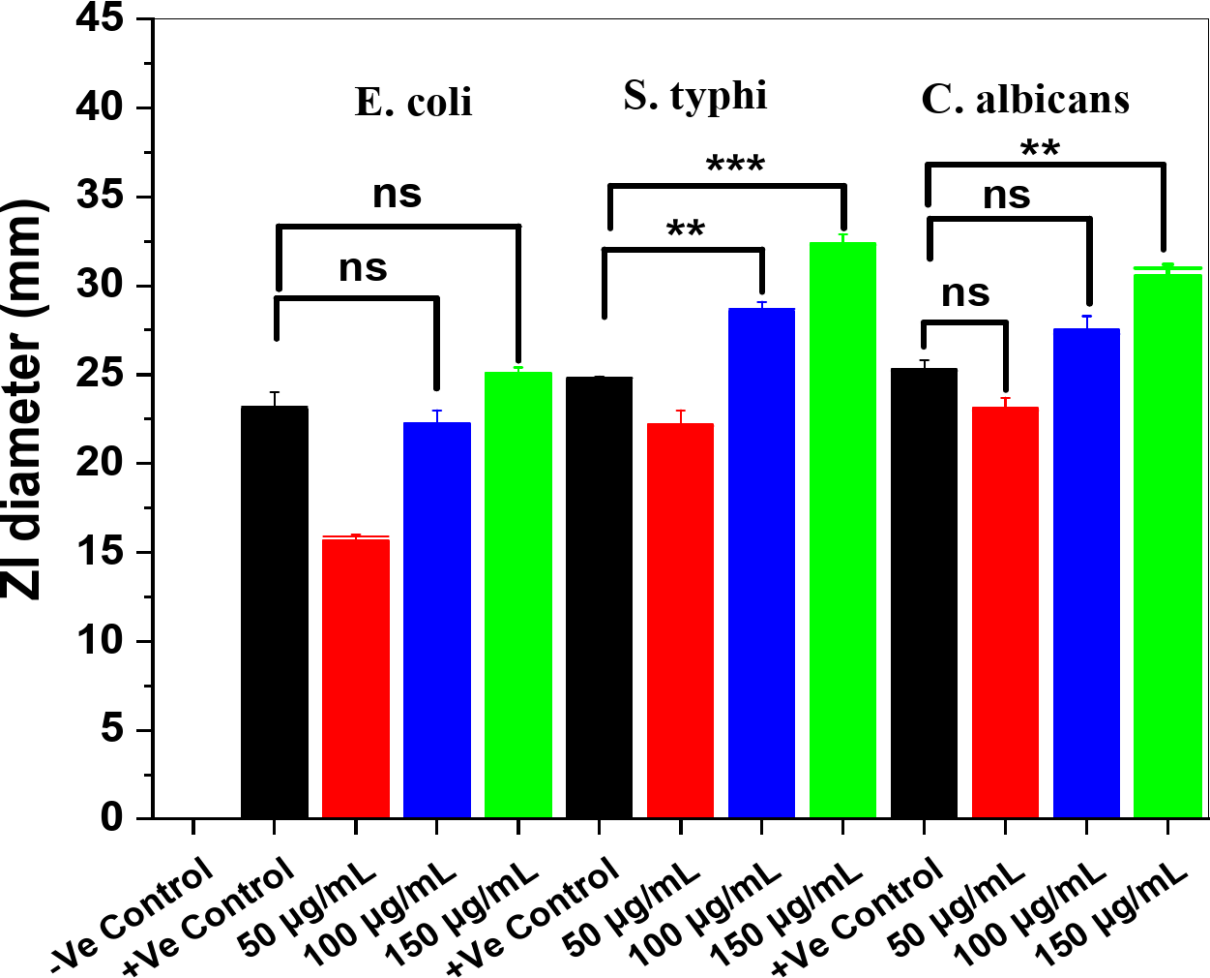
The ZI diameters with means ± SDs (in mm) of the investigated pathogenic microbes *E. coli* and *S.typhi* (Gram -Ve), and *C. ablicans* against various concentrations (50, 100, and 150 µg/mL) of ZnO/ZnS nanocomposite. *p < 0.05, **p < 0.01, ***p < 0.001 (*n* = 3).

Generally, the maximum ZI diameter was estimated against both *MRSA* and *B. subtilis* bacteria (Fig. 11) at 150 µg/mL and was 35 ± 0.21 (*p* < 0.001) and 33 ± 0.21 ± 0.32 mm (*p* < 0.001), respectively. These findings were compared with those reported by Hefny et al.^82^; Rai et al.^83^. However, the maximum ZI diameters (Fig. 12) for *E. coli* and *S. typhi*,* C. albicans* were 25 ± 0.19, 32 ± 0.36 (*p* < 0.001), and 31 mm (*p* < 0.01), respectively^62^. These findings were comparable when compared to results obtained by Abdo et al.^84^ and Abd El-Nour et al.^85^.

**Fig. 13:**
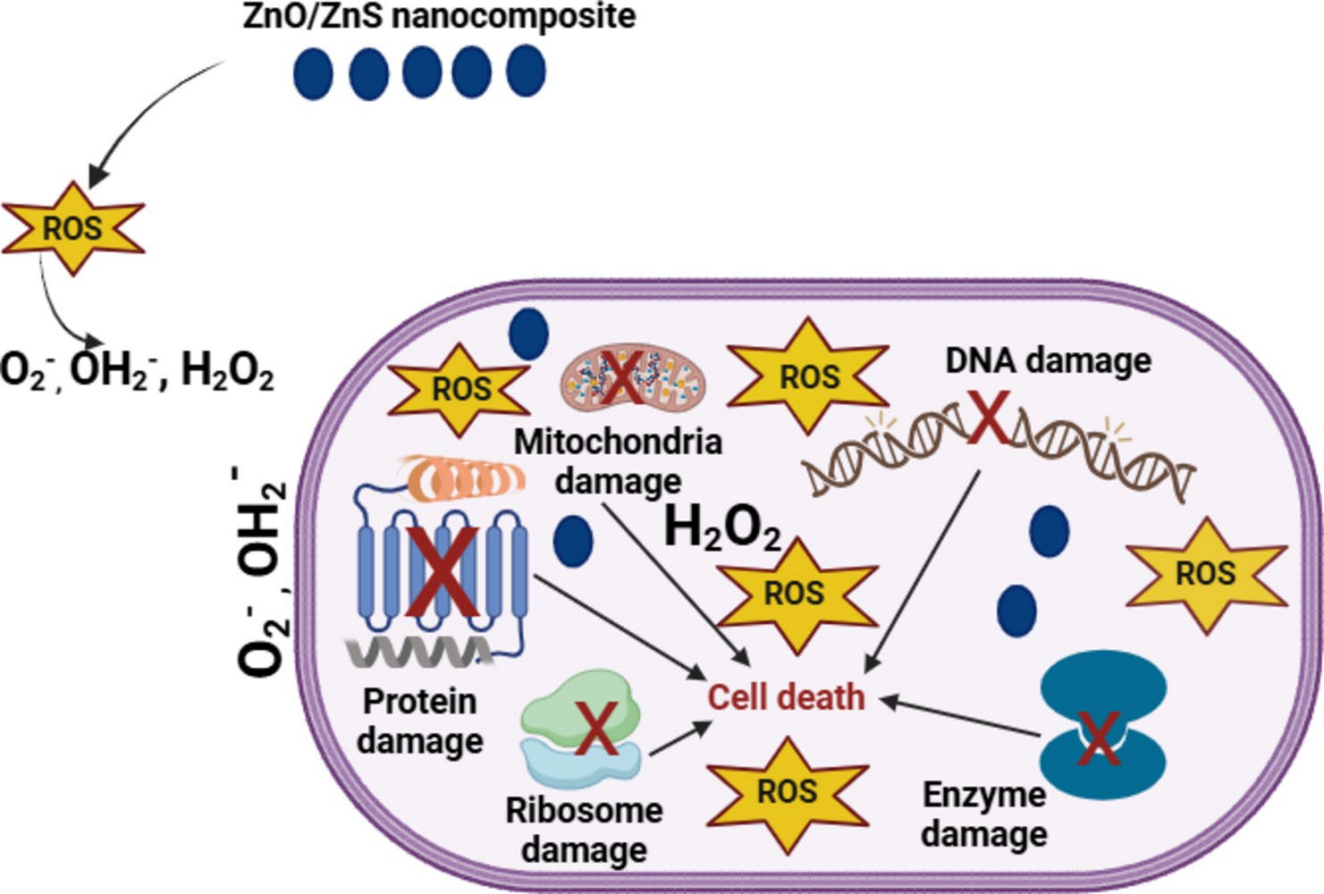
Suggested mechanisms of action of ZnO/ZnS nanocomposite against bacteria.

The proposed mechanism suggests that ZnO/ZnS nanocomposite release Zn^2+^ ions, which can be internalized into the bacterial cell and disrupt the enzymatic system (Fig. 13). Then, reactive oxygen species (ROS) production (causing the destruction of cellular components such as DNA, proteins and lipids): O_2_^−^ and HO_2_^−^ (do not penetrate the membrane, but direct contact causes damage) and H_2_O_2_ (internalised). Finally, internalisation within the bacteria cell and direct contact cause damage such as the loss of cellular integrity^86^.

Gram-negative bacteria have a thin peptidoglycan layer located between two membranes, which is recognized for giving antimicrobial resistance. Notably, dissociated carboxyl groups in the membranes provide negative charges on the cell surface. In contrast, ZnO NPs possess a positive charge, with a zeta potential of + 24 mV^87^. This difference creates a potent electrostatic interaction, which can disrupt the cell membrane due to the electrostatic gradient between the negatively charged membrane and the positively charged Zn²⁺ ions. As a result, both *E. coli* and *S. typhi* exhibit inhibition even at minimal concentrations of the ZnO/ZnS nanocomposite, showcasing the promising potential of this approach in combating serious bacterial infections^86^.

The quantity of tested NPs that were primarily used strongly affected the estimated inhibition zone diameter. Therefore, it is imperative to evaluate the MIC value for the biosynthesized ZnO/ZnS nanocompositeagainst each investigated pathogen. This was ascertained by detecting the lowest concentration at which bacterial growth is inhibited^88,89^. Various concentrations of ZnO/ZnS nanocomposite ranging from 50 to 50,000 µg/mL were utilized.

These results revealed that the MIC values of ZnO/ZnS NPs for *B. subtilis* and *MRSA* were 50,000 and 500 µg/mL, respectively. On the other hand, the values of MIC for *E. coli* and *S. typhi* have been estimated to be 5,000 and 50,000 µg/mL, respectively. Thus, from the observations, it is conclusive that the bimetallic nanostructure ZnO/ZnS is more effective on both *MRSA* and *E. coli*. Thus, it is more effective against *E. coli* than against *B. subtilis* and *S. typhi*. Applications of ZnO/ZnS nanocompositehave efficiently inhibited growth among harmful pathogenic bacterial strains.

### SEM bacterial cell imaging

**Fig. 14:**
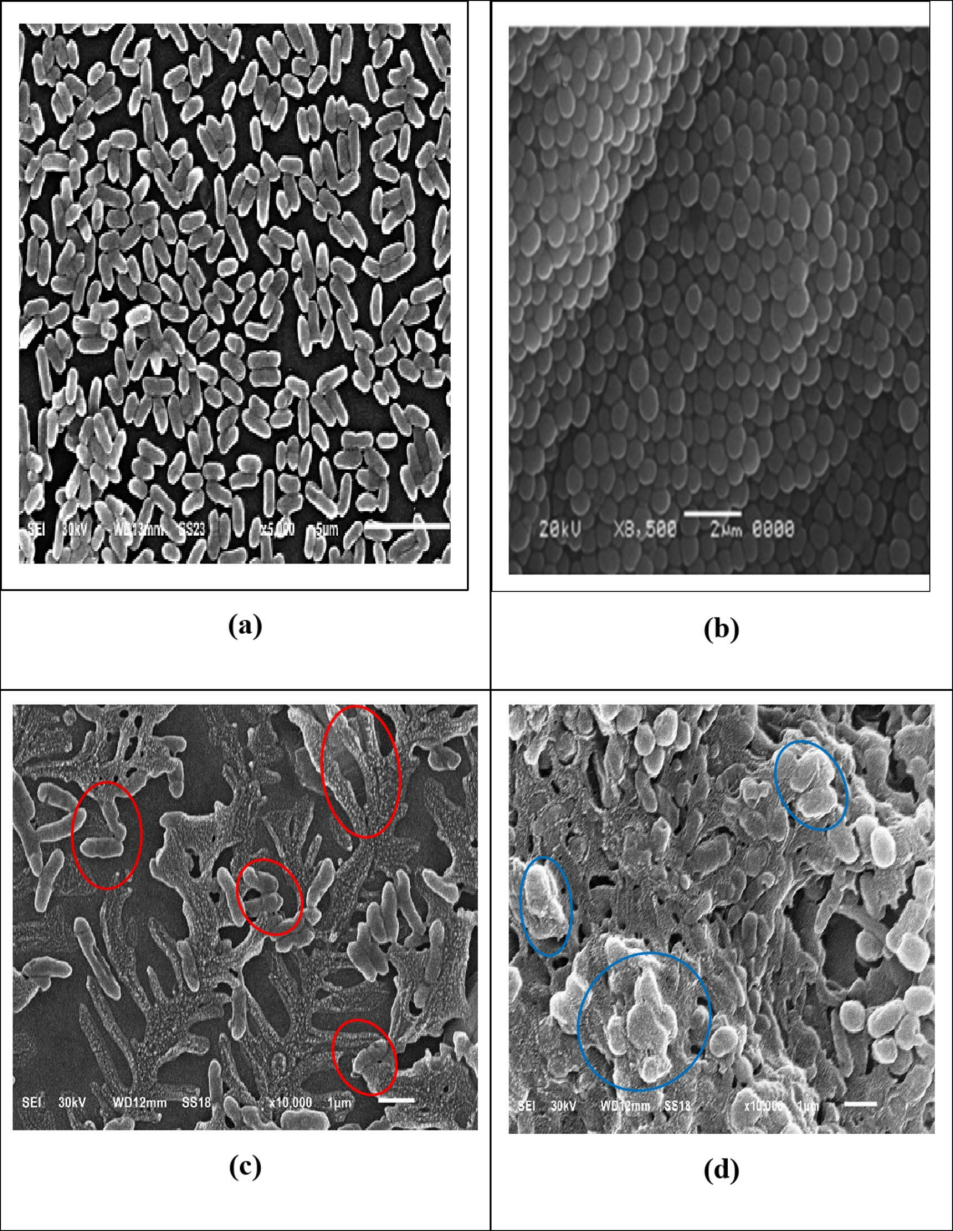
SEM micrographs of bacterial samples (**a**) control *E. coli*, (**b**) control *MRSA*, (**c**) *E. coli* treated with ZnO/ZnS nanocomposite (below-MIC 500 µg/mL), and (**d**) *MRSA* treated with ZnO/ZnS nanocomposite (below-MIC µg/mL) at 5000X magnification.

SEM imaging is an appropriate technique for examining the microstructure and morphology of bacterial cells. Figure 14a–d show SEM micrographs of both treated and untreated bacterial cells. SEM analysis was used to investigate the structural alterations in the outer membrane of the untreated cells compared with treated cells with the nanocomposite. Figure 14a displays a representative SEM picture of *E. coli* cells (normal cells) before the incorporation of the biosynthesized ZnO/ZnS nanocomposite based on their antibacterial activity. It can be noticed that the surfaces of rod-like bacteria were smooth and damageless (red circles). This suggests that before treatment with nanoparticles, the cells were normal. However, post-treatment ZnO/ZnS nanocomposite exhibited surface wrinkling and a significant degree of deterioration, as illustrated in Fig. 14c. (blue circles). Therefore, it is presumptively believed that the antimicrobial effects are a result of the interactions between the ZnO/ZnS nanocomposite and the bacterial cell membrane (Fig. 14d). The NPs pass through the cell and ultimately impact numerous target areas^90,91^. Moreover, undesirable or external chemicals can disrupt the cellular membrane, compromising its integrity and impairing the operative capacity of the barrier’s capacity to operate ultimately leading to cell death^92–94^.

### MTT cell cytotoxicity assay

**Fig. 15:**
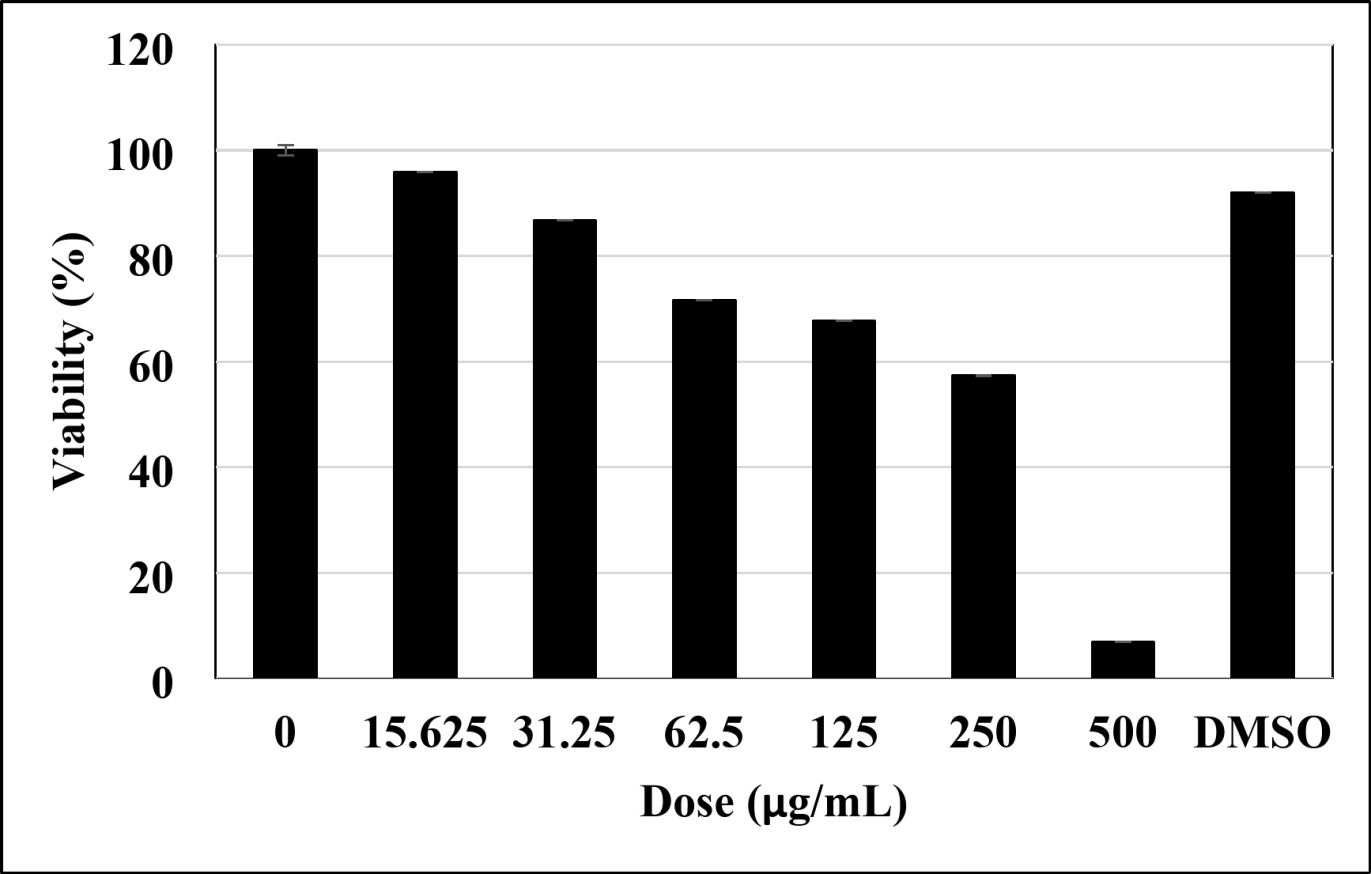
Bar diagram of the in vitro cytotoxic effects of the ZnO/ZnS nanocomposite on the MDA-MB-231 cancer cell line.

**Fig. 16:**
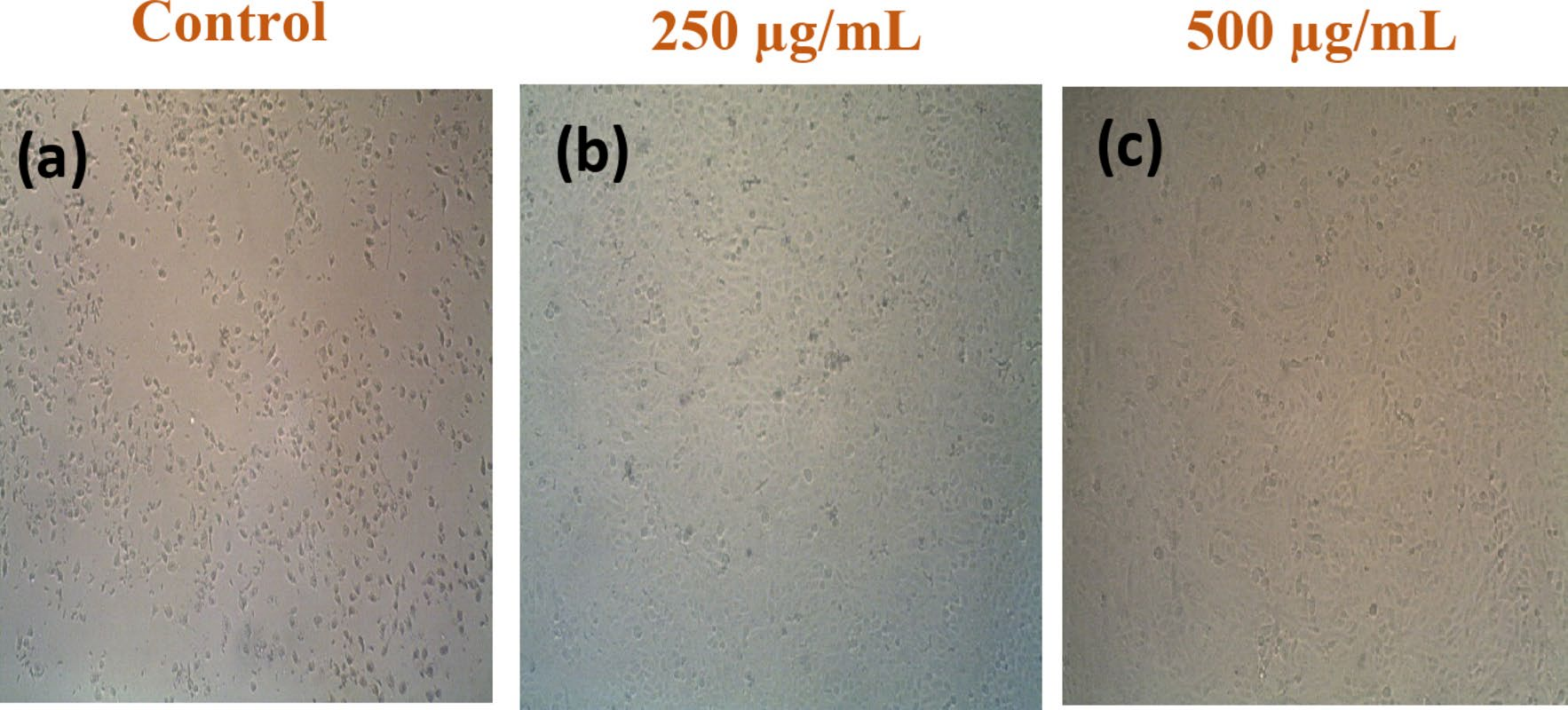
Light inverted micrographs showing morphological changes in the exposed sample of the MDA-MB-231 cell line (**a**) and at different concentrations of ZnO/ZnS nanocomposite (**b**) 250 and (**c**) 500 µg/mL.

The MTT assay is a standard methodology employed in toxicology to elucidate the cellular response to a harmful substance and is commonly referred to as a viability assay. With regard to cell proliferation and cytotoxicity studies, the MTT assay is an extremely sensitive colorimetric technique that is utilized to evaluate the viability of cells. This was performed using the MDA-MB-231 cancer cell line^95^. Furthermore, Mahendiran et al.^96^, and Saranya et al.^97^ offer comprehensive insights into metabolic processes, cellular viability, and apoptosis. Figure 15 shows the correlation between the mortality rate of cancer cells and the dosage of ZnO/ZnS nanocomposite, as observed at different concentrations. The results demonstrated that the IC_50_ value for the MDA-MB-231 cancer cell line was reported to be 197 ± 0.895 µg/mL. The findings suggested that the viability of the MDA-MB-231 cancer cell line decreased with increasing concentrations (Fig. 15) and durations of exposure to ZnO/ZnS nanocomposite. Figure 16 displays the effect of the nanocomposite on the viability of the MDA-MB-231 cells. It can be noticed that the number of cells significantly decreased with increasing the nanocomposite concentration. The capacity of the nanocomposite to form reactive oxygen species (ROS) may be the cause of this phenomena^97^. Since the elevated levels of ROS (Fig. 13) will severely damage cellular DNA, slowing the cell cycle and ultimately causing cell death^98–101^.

### MB dye degradation

**Fig. 17:**
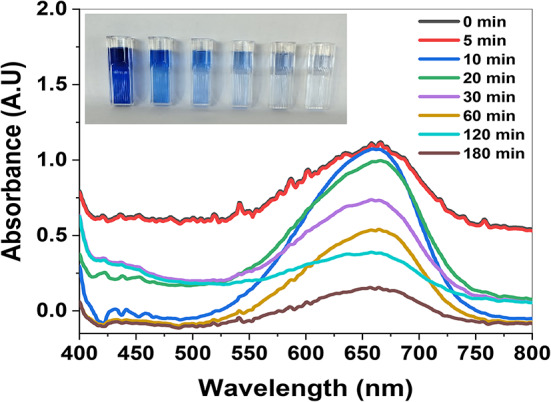
UV–Vis absorbance spectra of MB dye in the presence of the bioreduced ZnO/ZnS nanocomposite catalyst under dark conditions (inset shows photographs of MB dye degradation at different times).

**Fig. 18:**
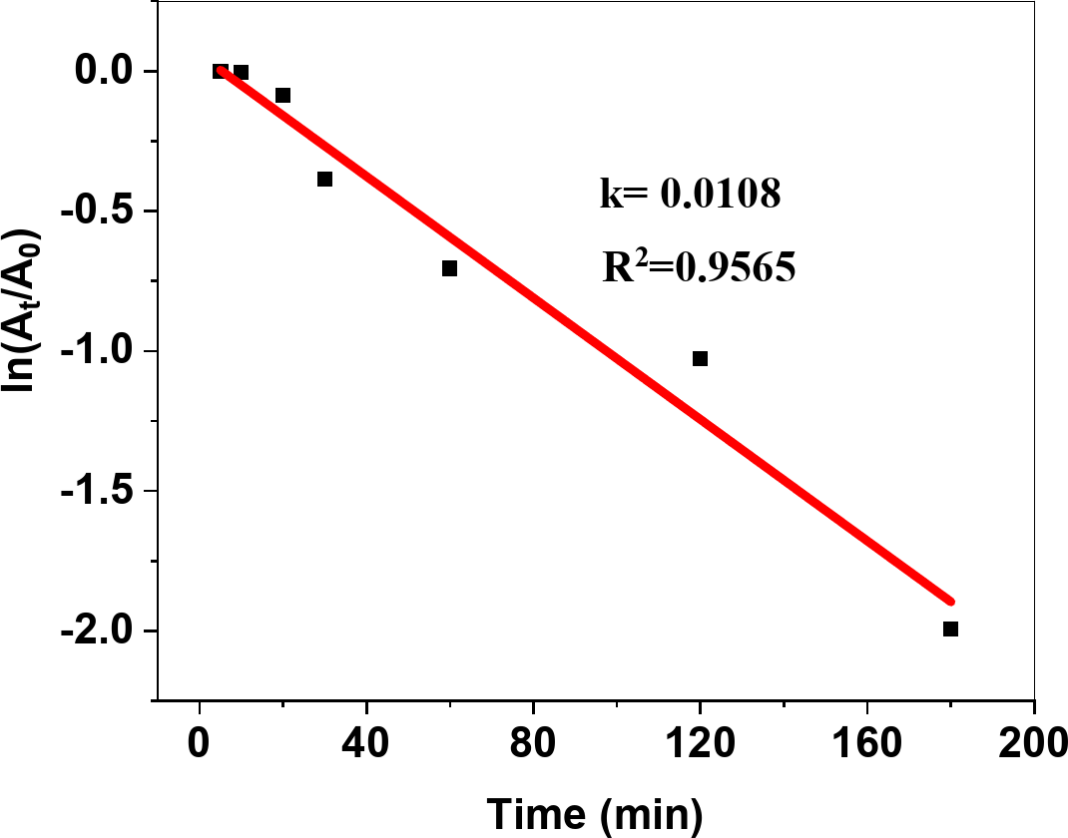
ln(A_t_/A_0_) vs. time plots of MB dye degradation with and without the ZnO/ZnS nanocomposite.

The catalytic degradation profile of MB dye in the dark was assessed by using the spectrophotometric analysis approach. At the beginning of UV–Vis absorbance, the detected maximum absorption peak of the MB dye was observed at λ_max_ = 664 nm^102^. The dye’s absorbance readings were recorded at intervals of 0, 5, 10, 20, 30, 60, 120, and 180 min, respectively. It can be noticed that the behavior declined as the reaction time increased up to 180 min in the presence of the ZnO/ZnS nanocomposite catalyst under dark conditions (Fig. 17). The detected decrease indicates a drop in the original concentration of the MB dye. The natural logarithm graph in Fig. 18 depicts the ratio of the final concentration of MB dye to its initial concentration (ln(A_t_/A_0_)) as a function of time for degradation reactions facilitated by the catalysis of bioreduced ZnO/ZnS nanocomposite to the dye. The investigated reaction exhibits a correlation with pseudo-first-order kinetics, enabling the calculation of the rate constant (*k*) of the catalytic process using the subsequent equation:

2 $$\:ln\frac{{C}_{t}}{{C}_{0}}\:=-kt\:\text{o}\text{r}\:ln\frac{{C}_{t}}{{C}_{0}}=\:ln\frac{{A}_{t}}{{A}_{0}}=-kt$$

Here, *C*_*0*_ and *C*_*t*_ denote the concentrations of MB dye before and after the catalytic reaction, respectively.

In addition, Fig. 17 displays a graph illustrating the effectiveness of bioreduced ZnO/ZnS nanocomposite catalyst in removing MB dye without light. The initial concentration of the MB dye was denoted (C_0_). Subsequently, the variation in the MB (C_t_) concentration was quantified at certain time intervals of 0, 5, 10, 20, 30, 60, 120, and 180 min. At the same time, the catalytic reaction solution, including MB dye and ZnO/ZnS nanocomposite, was treated to darkness. The catalytic efficiency of the ZnO/ZnS nanoparticles for the breakdown of MB dye was determined using the equation specified below:

3 $${\text{Catalytic efficiency }}\left( \% \right){\text{ }}={\text{ }}\left( {{C_0} - {C_t}/{C_0}} \right) \times {\text{1}}00$$

**Fig. 19:**
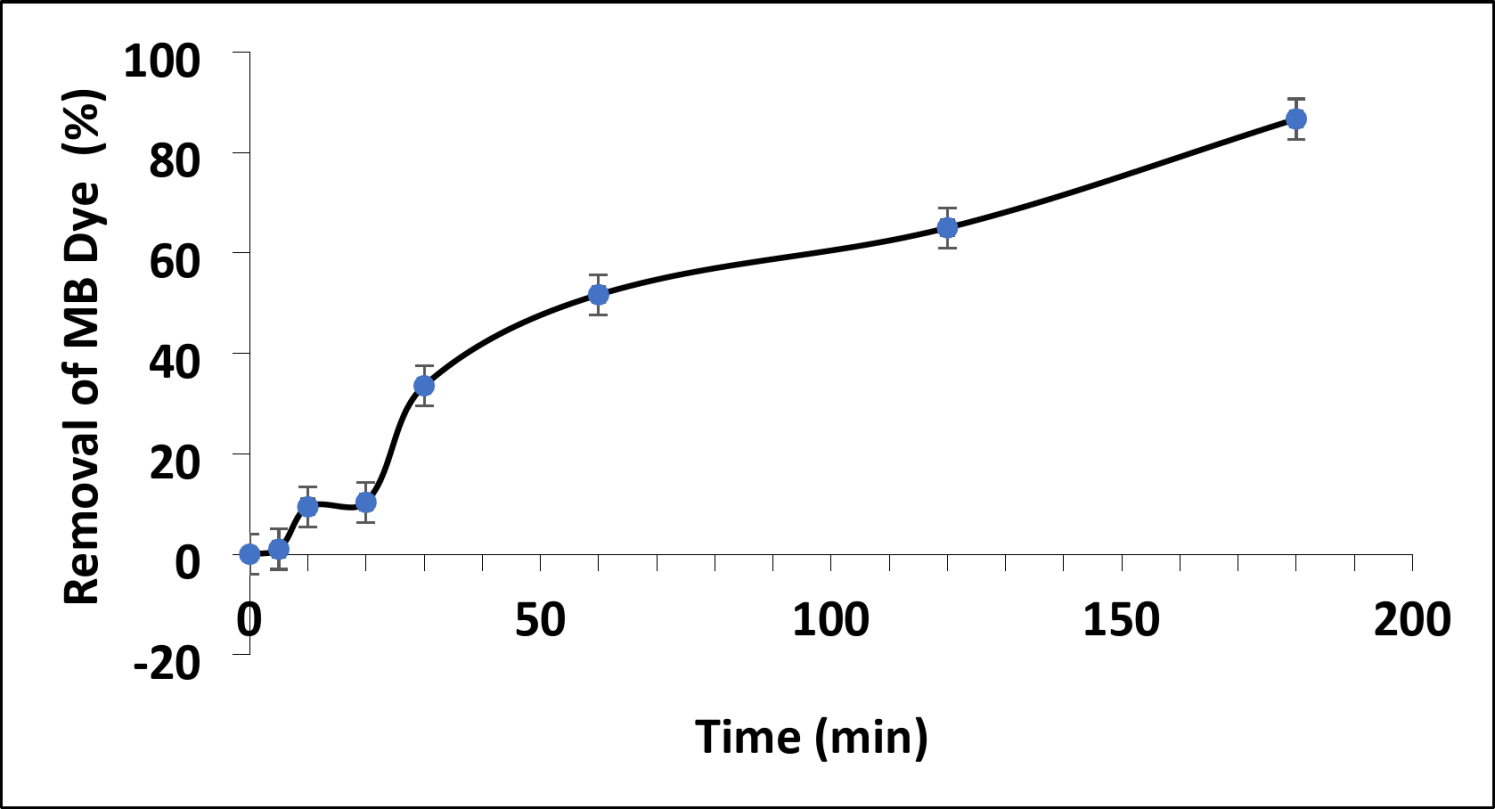
Plot of MB dye removal efficiency by the ZnO/ZnS nanocomposite under dark conditions.

The catalytic reduction reaction of the MB dye using ZnO/ZnS nanocomposite follows the Langmuir‒Hinshewood (LH) model^103^. The plots of ln(*A*_*t*_/*A*_0_) versus time yielded good linear correlations (Fig. 17), showing that the MB dye catalyzed by the ZnO/ZnS nanocomposite followed first-order kinetics. Since the rate of the equation can be written as $\:ln\frac{{A}_{t}}{{A}_{0}}=-kt$, Where *k* is the rate constant of the first-order kinetics, *A*_0_ is the initial concentration of MB dye, and *A*_*t*_ is the concentration at time *t*. The observed values of the first-order rate constant (*k*) and correlation coefficient (R^2^) for the ZnO/ZnS nanocomposite prepared via the green synthesis route were 1.0 × 10 –2 s − 1 and 0.9565, respectively. Furthermore, the concentration of MB dye achieved stability after 180 min of adsorption. This indicates that both the MB dye molecules and the ZnO/ZnS nanocomposite reached a state of equilibrium between adsorption and desorption at 180 min. Consequently, 87.61% of the MB dye was eliminated during the adsorption process (Fig. 19) in the presence of the bioreduced ZnO/ZnS nanocomposite catalyst.

Furthermore, electrostatic interactions may facilitate the adsorption of positively charged MB dye molecules onto negatively charged ZnO/ZnS nanocomposite surfaces. Heger et al.^104^ have suggested that the MB dye’s n to π* transitions are responsible for the maximum absorption peak observed at 664 nm. Moreover, the ZnO/ZnS nanocomposite catalyst markedly reduced the concentration of MB dye in an exponential manner, leading to the removal of 87.61% of the MB dye throughout the 180-minute catalysis process^102^. The present study suggests that the photocatalytic activity reported can be ascribed to the ZnO nanocatalyst’s tiny particle size, excellent crystallinity, and adequate bandgap energy^105^.

## Conclusion

This work reported the novel synthesis of ZnO/ZnS nanocomposite using CFF of *F. oxysporum*. The nanocomposite was synthesized at a room temperature of 27 ± 1.0 °C. The successful production of ZnO/ZnS nanocomposite was confirmed by characterization studies, which included UV‒Vis spectroscopy, FTIR, XRD, EDX, and TEM techniques. TEM imaging results indicated that the ZnO NPs had a nearly spherical shape with adjacent ZnS NPs layer. The dimensions of these NPs varied between 15 and 80 nm, as quantified using the DLS technique. Moreover, the FTIR analysis revealed several changes in the intensity and position of the peaks in the spectrum of the ZnO/ZnS nanocomposite compared to the fungal CFF. The observed changes may be ascribed to the physical interaction between the fungal biomolecules and the ZnO/ZnS nanocomposite, which functions as both a reducing and capping agent. This study evaluated the effectiveness of the nanostructures in terms of their antibacterial activity against several pathogenic bacterial isolates and their catalytic efficiency in eliminating the MB dye. Furthermore, the results indicate that the ZnO/ZnS nanocomposite displayed significant antibacterial effects against *B. subtilis* and *MRSA* bacteria, as shown by their minimum inhibitory concentration values of 50,000 and 500 µg/mL, respectively. However, the MIC values for *S. typhi* and *E. coli* have been estimated to be around 5,000 and 50,000 µg/mL, respectively. SEM images confirm the bacterial cell membrane damage according to the ROS species formation due to the NPs stress effect. Moreover, the nanocomposite demonstrated cytotoxic properties against the MDA-MB-231 cell line with the IC_50_ of 197 ± 0.89 µg/mL. Throughout an adsorption period of 180 min, the stability of the MB dye degradation was observed, indicating that both the MB dye molecules and the ZnO/ZnS NPs achieved a state of adsorption-desorption equilibrium. The results suggest that the biosynthesized ZnO/ZnS nanocomposite is strongly recommended for use in various biological, industrial, and biomedical application fields.

## Data Availability

The datasets used and/or analysed during the current study available from the corresponding author on reasonable request.
